# Shentong Zhuyu Decoction Inhibits Inflammatory Response, Migration, and Invasion and Promotes Apoptosis of Rheumatoid Arthritis Fibroblast-like Synoviocytes via the MAPK p38/PPAR*γ*/CTGF Pathway

**DOI:** 10.1155/2021/6187695

**Published:** 2021-01-15

**Authors:** Ying Han, Jing Wang, Meng Jin, Lin Jia, Cuihuan Yan, Yali Wang

**Affiliations:** ^1^College of Integrated Chinese and Western Medicine, Hebei Medical University, Shijiazhuang, China; ^2^Department of Chinese Medicine, The Third Hospital of Hebei Medical University, Shijiazhuang, China; ^3^Department of Chinese Medicine Diagnostics, Hebei University of Chinese Medicine, Shijiazhuang, China; ^4^Department II of Respiratory, Hebei Provincial Hospital of Traditional Chinese Medicine, Shijiazhuang, China; ^5^Institute of Integrated Chinese and Western Medicine, Hebei University of Chinese Medicine, Shijiazhuang, China

## Abstract

**Introduction:**

The current study is aimed at exploring the effect of Shentong Zhuyu Decoction on the proliferation, migration, invasion, and apoptosis of rheumatoid arthritis fibroblast-like synoviocytes (RA-FLS) and its underlying molecular mechanism.

**Materials and Methods:**

The type II collagen-induced arthritis (CIA) model was established. Subsequently, the RA-FLS were isolated from the CIA rat model and identified by immunohistochemistry. The viability, apoptosis, cell cycle, migration, and invasion of RA-FLS were detected by the cell counting kit 8 (CCK-8) assay, flow cytometry, wound-healing assay, and transwell invasion assay, respectively. The levels of MAPK p38, PPAR*γ*, CTGF, Bcl-2, Bax, caspase-3, IL-1*β*, MMP-3, CDK4, and cyclin D1 were determined by qRT-PCR and western blotting, respectively.

**Results:**

After treatment with Shentong Zhuyu Decoction medicated serum, the OD_570_ value, migrative and invasive abilities, and the secretion of IL-1*β*, MMP-3 were remarkably decreased in RA-FLS, while the apoptosis rate was increased. Further, results showed that Shentong Zhuyu Decoction inhibited the transition from the G1 phase to S phase. Additionally, Shentong Zhuyu Decoction significantly inhibited the expression of Bcl-2, CDK4, cyclin D1, MAPK p-p38, and CTGF, whereas elevated the levels of Bax, caspase-3, and PPAR*γ*. Importantly, the effects of Shentong Zhuyu Decoction were consistent with the trends of MAPK P38 inhibitor (SB203580) and PPAR*γ* agonist (GW1929).

**Conclusions:**

Shentong Zhuyu Decoction inhibited viability, inflammatory response, migration, invasion, and transition from the G1 phase to S phase and promoted apoptosis of RA-FLS via the MAPK p38/PPAR*γ*/CTGF pathway.

## 1. Introduction

Rheumatoid arthritis (RA) is a chronic autoimmune disease with high prevalence and disability, which involves in multiple joints throughout the body [1, 2]. Currently, the treatment of RA is mainly based on drugs and surgeries [3]. However, high costs seriously trouble the patients, and treatments are far from meeting the needs of RA patients. Furthermore, the pathology of RA is still unclear [4]. As we known, fibroblast-like synoviocytes (FLS) show tumor-like characteristics, which are considered to be the “leader cells” in terms of promoting the persistence and destructiveness in disease. Moreover, FLS play a major role on recurrence, long-term cure, and poor prognosis of RA [5, 6]. Previous research has found that targeted treatment for FLS improves the condition of RA and indicated that direct regulation of FLS may become the next target for RA treatment [7]. Therefore, exploring the underlying mechanism and seeking for new therapy are urgent for public health.

Previous study has indicated that the mitogen-activated protein kinase (MAPK) family members play vital roles on progression of RA including MAPK p38, extracellular signal-regulated kinase (ERK), and c-Jun N-terminal kinase (JNK). In particular, MAPK p38 is involved in the upregulation of proinflammatory cytokines [8]. Moreover, the MAPK p38 pathway has been proved to participate in the activation of RA-FLS [9, 10]. Peroxisome proliferator-activated receptors (PPARs) are known as ligand-activated transcription factors. In terms of RA, a previous study has demonstrated that activation of PPAR*γ* can effectively suppress the inflammatory response [11]. In addition, connective tissue growth factor (CTGF) is a downstream molecule of PPAR*γ* [12] and plays a critical role on RA progression, which is verified, respectively [13]. Furthermore, a previous study has indicated that activation of MAPK p38 negatively regulates PPAR*γ* [14]. Therefore, we hypothesized that the MAPK p38/PPAR*γ*/CTGF pathway may involve in RA progression.

More and more evidence have demonstrated that traditional Chinese herbs including liquiritin, berberine, salicin, imperatorin, and paeonol play vital roles on the proliferation, inflammation, and angiogenesis in the RA [15–19]. Furthermore, Duan et al. [20] have indicated the synergistic mechanism of four effective components of compound Fengshiding capsule against RA by network pharmacology, which may provide basis for the further study on compound preparation of traditional Chinese medicine. Shentong Zhuyu Decoction consists of about 12 kinds of traditional Chinese medicine, which plays roles on promoting blood circulation and removing blood stasis [21]. Moreover, previous studies have demonstrated that Shentong Zhuyu Decoction inhibits the inflammatory response and alleviates the pain behaviors [22, 23]. However, whether Shentong Zhuyu Decoction regulates the molecular mechanism of RA-FLS via the MAPK p38/PPAR*γ*/CTGF pathway has not reported yet.

The current study is aimed at exploring the effect of Shentong Zhuyu Decoction on the proliferation, migration, invasion, and apoptosis of RA-FLS and its underlying molecular mechanism via the MAPK p38/PPAR*γ*/CTGF pathway. Our findings may provide the novel therapy for treated with RA.

## 2. Materials and Methods

### 2.1. Animal Model

Sprague-Dawley (SD) rats were purchased from Liaoning Changsheng Biotechnology Co., Ltd. (Shenyang, China). The collagen-induced arthritis (CIA) rat model was established as previously described [24]. Briefly, type II collagen (CII, 10 mg) was dissolved in acetic acid (0.1 mol/L) and then mixed with an equal amount of complete Freund's adjuvant (CFA). Subsequently, rats were injected with CII emulsion (100 *μ*L) in the root of the tail, respectively. After 7 days, the immunization was repeated again with the same dosage in the same site. The rats were injected with normal saline of equal volume, which is deemed as control. On the 14^th^ days of modeling, a marked swelling of the toe and ankle joint was observed.

The arthritis scores of CIA rats were evaluated by the scoring criteria [25]. In brief, 0 point indicated no erythema and swelling, 1 point indicated mild erythema and swelling of the mid-foot or ankle joint, 2 points indicated mild erythema and swelling extending from the ankle to mid-foot, 3 points indicated moderate swelling extending from the ankle to the metatarsal joints, and 4 points indicated that severe swelling encompass the ankle, foot, and digits. The maximum arthritic score per rats was 16. In general, arthritis scores > 4 are deemed as an indicator for successful modeling [26]. The paw swelling of CIA rats was assessed by measuring the circumference of the paw, as previously described [27]. Oedema was expressed in percentage compared with the initial control values.

The parameters of the CIA model in the current study indicated that the levels of and rheumatoid factor (RF) and C-reactive protein (CRP) were (67.43 ± 13.95) IU/mL and (6.72 ± 1.66) mg/L, paw swelling was (21.44 ± 6.00%), and arthritis score was 8.00 ± 1.41. So, the rats with abovementioned parameters were regarded as the successful CIA rat model and then were used for subsequent experiments.

### 2.2. Prepare the Shentong Zhuyu Decoction

The Shentong Zhuyu Decoction consisted of Gentiana macrophylla (3 g), Ligusticum wallichii (6 g), Peach Kernel (9 g), Safflower (9 g), Licorice (6 g), Notopterygium (3 g), Myrrh (6 g), Angelica (9 g), Trogopterus dung (6 g), Rhizoma Cyperi (3 g), Achyranthes (9 g), Earthworm (6 g), Cortex phellodendri (6 g), and Rhizoma Atractylodis (6 g). Abovementioned herbs were Chinese medicine dispensing granules, which were purchased from Shenwei Pharmaceutical Group Co., Ltd. (Beijing, China). 1 dose of Chinese medicine dispensing granules was equivalent to 87 g of crude drug, which added boiling water (55.55 mL) to make the drug concentration of 1.566 g/mL.

### 2.3. Medicated Serum Preparation

The dosage of Shentong Zhuyu Decoction was converted by the human and animal body surface area, determining by the common clinical dosage, which was about 15.66 g/kg/d. The normal rats were gavaged by Shentong Zhuyu Decoction (2 mL) and distilled water (2 mL) for 7 days, respectively. Two hours after the last administration, the rats were anesthetized, and 5 mL of blood was drawn from the abdominal aorta. The blood was centrifuged at 1000 × g for 10 min after stood for 30 min at room temperature. Following, the drug-containing serum and control serum were collected and stored at -80°C. Finally, the drug-containing serum was added into culture medium to detect the effect on RA-FLS, with volume fractions of 5% (low-dose group), 10% (medium-dose group), and 20% (high-dose group), respectively. The appropriate concentration of drug-containing serum was screened using the MTT assay.

### 2.4. Cell Isolation and Identification

The synovial tissues were taken from normal and CIA rats and cut and digested with DNase (0.15 mg/mL), hyaluronidase (0.15 mg/mL), and collagenase (1 mg/mL). The normal FLS (normal FLS) and RA-FLS were obtained and resuspended with DMEM medium (Gibco, Gaithersburg, MD, USA) containing 10% fetal bovine serum (FBS, Tianhang Biotechnology Co., Ltd, Hangzhou, China), 30 mg/mL glutamine (Solarbio, Shanghai, China), 2.5 *μ*g/mL amphotericin B (Sigma-Aldrich, St. Louis, Mo, USA), and 10 *μ*g/mL gentamicin (Gibco). After the overnight culture, the nonadherent cells were removed, and adherent cells were routinely cultured.

The RA-FLS were identified by immunohistochemistry. In brief, the cells (2 × 10^4^ cells/mL) were cultured in 12-well plate for 48 h. Subsequently, cells were fixed with 4% paraformaldehyde and incubated with 0.1% TritonX-100 and 3% hydrogen peroxide. After washed, anti-Vimentin (1: 200, Wanleibio, Shenyang, China) and anti-CD68 (1: 200, Affinity Biosciences, OH, USA) were added and incubated overnight at 4°C. Following, horseradish peroxidase- (HRP-) labeled goat anti-rabbit IgG was added and incubated overnight at 37°C for 60 min. Finally, the cells were stained with diaminobenzidine (DAB) and hematoxylin. The images were captured by inverted microscope (BX53, Olympus, Tokyo, Japan).

### 2.5. Cell Treatment and Grouping

The cells in the logarithmic phase of growth were collected and cultured in 96-well plates (5 × 10^3^ cells/mL). After the cells were adhered, different drugs were intervened for 48 h. The experiment was divided into 5 groups. Among them, normal FLS were cultured in DMEM medium plus 20% control serum (normal group). RA-FLS were cultured in DMEM medium plus 20% control serum, P38 mitogen-activated protein kinase inhibitor (MAPK P38 inhibitor, SB203580, 20 *μ*mol/L), peroxisome proliferator-activated receptor gamma agonist (PPAR*γ* agonist, GW1929, 30 *μ*mol/L), and 20% drug-containing serum, which were named as the blank group, P38 inhibitor group, PPAR*γ* agonist group, and Shentong Zhuyu Decoction group, respectively.

### 2.6. Cell Viability Assay

Cell viability was detected by the cell counting kit 8 (CCK-8) assay. Cells (5 × 10^3^ cells/mL) were seeded in 96-well plates and then intervened with drug for 48 h. CCK-8 solution was added into per well and incubated for 2 h. The OD_570_ values were measured by microplate reader (ELX-800, Biotek, Winooski, VT, USA).

### 2.7. Cell Apoptosis Assay

After 48 h treatment, FLS were collected, washed with cool phosphate buffer saline (PBS) and resuspended. The cell apoptosis was conducted by flow cytometry (NovoCyte, ACEA Bioscience, San Diego, California, USA) using Annexin V-FITC apoptosis detection kit (Wanleibio).

### 2.8. Cell Cycle Assay

After 48 h treatment, FLS were collected, fixed with 75% ethanol, and washed with cool PBS. Subsequently, RNA enzyme-containing propidium iodide (PI) solution was added for incubation. Finally, the cell cycle was detected by instrument (NovoCyte, ACEA Bioscience).

### 2.9. Wound-Healing Assay

After treatment, FLS were cultured in 6-well plates (1 × 10^5^ cells/mL) overnight. The wound was obtained by using a 200 *μ*L pipette tip on the center of the cell monolayer. Images were captured at 0 h and 48 h, and the migrative distance was assessed by Image pro plus software.

### 2.10. Transwell Invasion Assay

The cells (1 × 10^5^ cells/mL) were grown in the top chamber with Matrigel (BD Bioscience, Franklin Lakes, NJ, USA), and DMEM medium was added in the lower chamber. After 48 h, the invasive cells were fixed with 4% paraformaldehyde and the stained with 0.4% crystal violet (Amresco, Cleveland, OH, USA). Finally, the amount of cells was calculated via microscope (IX53, Olympus).

### 2.11. Quantitative Real-Time RT-PCR

Total RNA was extracted from cells using TRIpure isolation reagent (BioTeke, Beijing, China). qRT-PCR analysis was performed as previously described [28]. Briefly, first-strand cDNA was synthesized from total RNA using cDNA synthesis kit (BioTeke). Then, real-time PCR was performed using the SYBR Green kit (Solarbio) and detected by Exicycler TM 96 (Bioneer, Daejeon, Korea). GAPDH was acted as internal control. The primer sequences were synthesized by GenScript Biotechnology (Nanjing, China) and listed as the following: MAPK p38 forward, TAAAGCCCAGCAACCTCG, reverse, CAGCCCACGGACCAAATA; PPAR*γ* forward, TACCACGGTTGATTTCTC, reverse, AATAATAAGGCGGGGACG; CTGF forward, GTCTTCGGTGGGTCCGTGTA, reverse, GCGGTCCTTGGGCTCATCAC; CyclinD1 forward, GAGGAGCAGAAGTGCGAAGA, reverse, GGCGGATAGAGTTGTCAGTGTAG; caspase-3 forward, GACGACAGGGTGCTACGAT, reverse, TTTCCTTACGCTCTGACTGA; *β*-actin forward, GGAGATTACTGCCCTGGCTCCTAGC, reverse, GGCGGACTCATCGTACTCCTGCTT. The reaction was conducted with an initial denaturing step at 94°C for 5 min, followed by 40 cycles of 94°C for 10 s, 60°C for 20 s, and 72°C for 30 s. The relative expression was analyzed by the 2^-*ΔΔ*Ct^ method.

### 2.12. Western Blotting

Protein samples were extracted by RIPA buffer, and the concentration was measured by the BCA kit. Protein was separated by SDS-PAGE and then was transferred into polyvinylidene difluoride (PVDF) membrane. After that, membranes were blocked with 5% skim milk, incubated overnight at 4°C with monoclonal anti-*β*-actin antibody, anti-p38 antibody, anti-p-p38 antibody, anti-CTGF antibody, anti-MMP-3 antibody, and anti-IL-1*β*antibody (1: 1000, Wanleibio), and then incubated with the horseradish peroxidase- (HRP-) conjugated secondary antibody (1: 10000, Wanleibio) for 50 min. Immunodetection was performed using enhanced chemiluminescence reagent (Wanleibio), and then the protein band was detected using Gel-Pro-Analyzer program (Media Cybernetics, Rockville, MD, USA). *β*-actin was used as internal control.

### 2.13. Statistical Analysis

All data were statistically analyzed with SAS 9.1 software. The measurement data were expressed as mean ± standard deviation (SD). The differences between two groups were analyzed by the *t*-test. The comparison of multiple groups was calculated with one-way analysis of variance (ANOVA) and followed by the LSD *t*-test. *P* < 0.05 was considered as statistically significant.

## 3. Results

### 3.1. RA-FLS Identification

**Figure 1:**
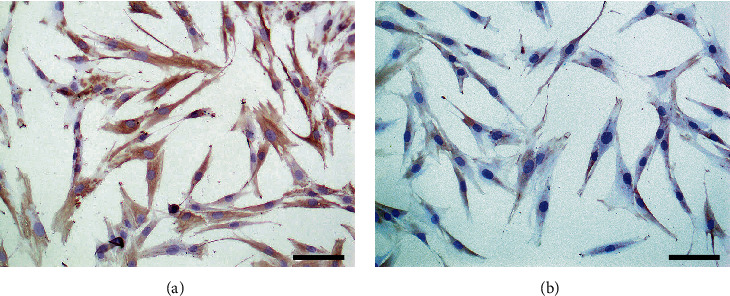
The rheumatoid arthritis fibroblast-like synoviocytes (RA-FLS) were identified by immunohistochemistry. (a) The results of immunohistochemistry showed that anti-Vimentin antibody was positive (×200). (b) The results of immunohistochemistry showed that anti-CD68 antibody was negative (×200).

After cultured 3 generations, RA-FLS presented typically fibroblast-like morphology, which was characterized by elongated spindle-shaped, elongated protrusions, nucleus-centered, round, or oval shape and arrangement of polarity. The results of immunohistochemistry showed that anti-Vimentin antibody was positive, and anti-CD68 antibody was negative (Figures 1(a) and 1(b)). Abovementioned results were in accordance with the characteristics of RA-FLS.

### 3.2. Screening of Concentration of Shentong Zhuyu Decoction Medicated Serum

**Figure 2:**
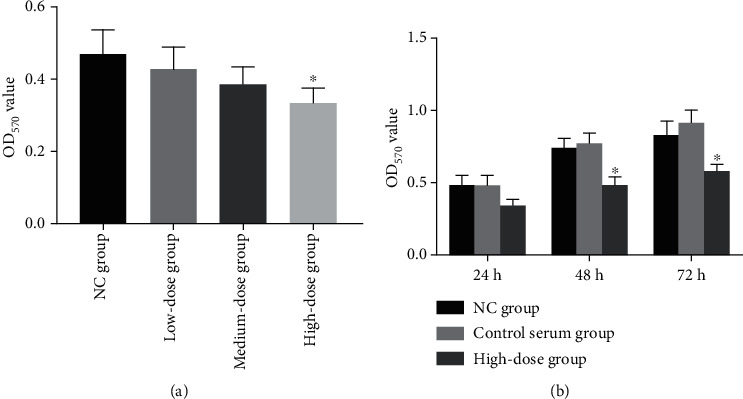
Screening of concentration of Shentong Zhuyu Decoction medicated serum. (a) The OD_570_ value of different drug-containing serums at 24 h intervention in RA-FLS. (b) The OD_570_ value of intervention with drug-containing serum at 24 h, 48 h, and 72 h in RA-FLS. NC group, without any addition in the culture medium; control serum group, culture medium plus 20% control serum.

MTT results showed that the OD_570_ value was significantly decreased in the high-dose group compared with the NC group (*P* < 0.05), indicating that intervention with 20% drug-containing serum for 24 h can be effectively suppressed the proliferation of RA-FLS (Figure 2(a)). Subsequently, the intervention time among different groups indicated that the OD_570_ value in the high-dose group at 24 h was slightly decreased, while not obviously different compared with the NC group. However, the OD_570_ value of the high-dose group at 48 h and 72 h was significantly lower than those of the NC group and control serum group (*P* < 0.05) (Figure 2(b)). Those findings suggested that 20% drug-containing serum treated for 48 h was selected as the intervention conditions for the subsequent study.

### 3.3. Shentong Zhuyu Decoction Inhibited Viability and Promoted Apoptosis of RA-FLS

**Figure 3:**
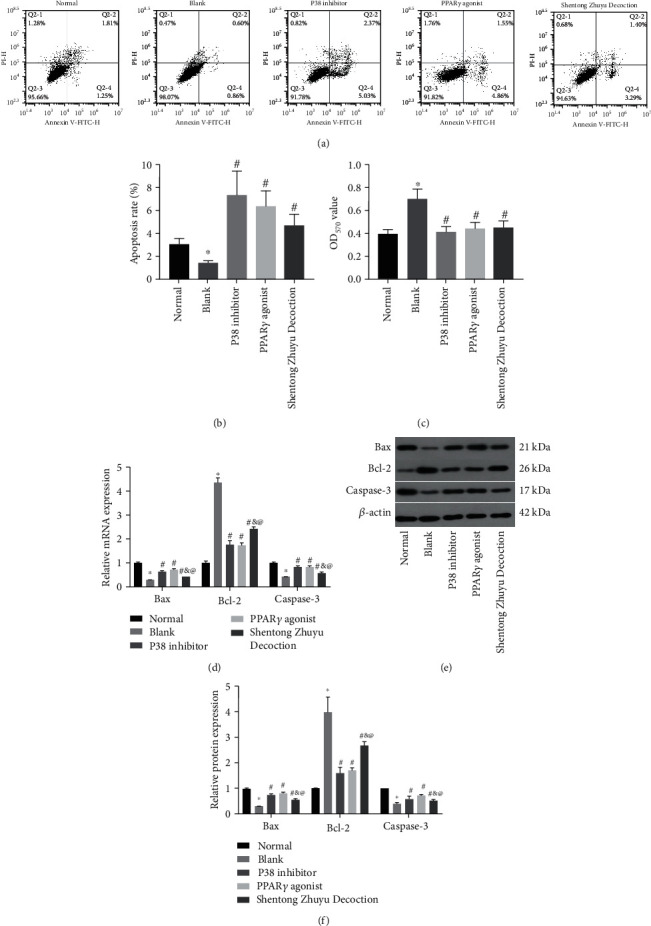
The effects of Shentong Zhuyu Decoction on RA-FLS. (a, b) The apoptosis rate of RA-FLS was detected by flow cytometry. (c) The OD_570_ value of RA-FLS was detected by the CCK-8 assay. (d) The mRNA levels of Bax, Bcl-2, and caspase-3 in RA-FLS were tested by qRT-PCR. (e, f) The protein levels of Bax, Bcl-2, and caspase-3 in RA-FLS were tested by western blotting. ^∗^*P* < 0.05 compared with the normal group, ^#^*P* < 0.05 compared with the blank group, ^&^*P* < 0.05 compared with the P38 inhibitor group, and ^@^*P* < 0.05 compared with the PPAR*γ* agonist group.

The results of flow cytometry showed that the apoptosis rate in the blank group was lower than that in the normal group (*P* < 0.05). In addition, the apoptosis rates in the P38 inhibitor group, PPAR*γ* agonist group, and Shentong Zhuyu Decoction group were remarkably increased compared with the blank group (*P* < 0.05) (Figures 3(a) and 3(b)). Moreover, CCK-8 assay results indicated that the trend of the OD_570_ value was contrary to that of the apoptosis rate (Figure 3(c)). All data of qRT-PCR and western blotting demonstrated that the levels of Bax and caspase-3 in the blank group were lower than those in the normal group (*P* < 0.05) and higher than in the P38 inhibitor group, PPAR*γ* agonist group, and Shentong Zhuyu Decoction group (*P* < 0.05). Meanwhile, the expression of Bcl-2 was opposite to that of Bax and caspase-3 (Figures 3(d)–3(f)). However, the OD_570_ value and apoptosis rate were not significant difference among the P38 inhibitor group, PPAR*γ* agonist group, and Shentong Zhuyu Decoction group (*P* > 0.05).

### 3.4. Shentong Zhuyu Decoction Influenced the Cell Cycle of RA-FLS

**Figure 4:**
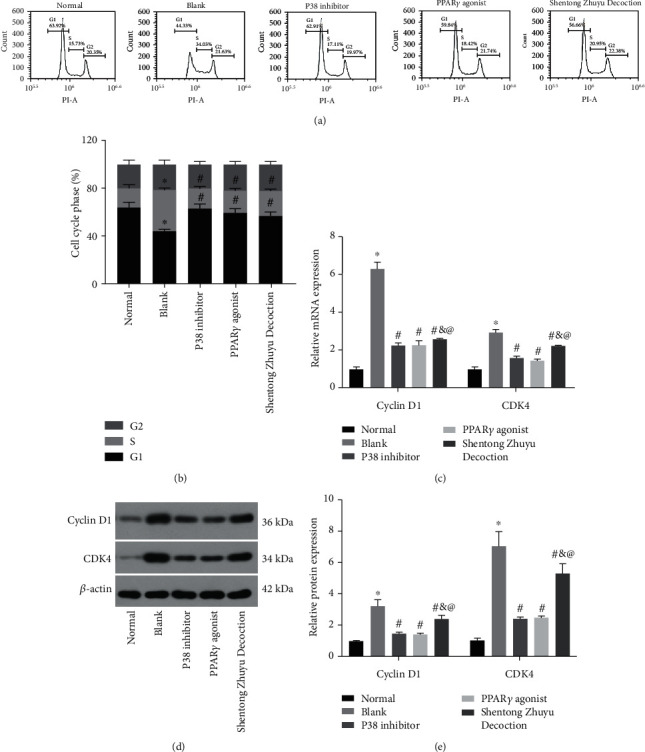
Shentong Zhuyu Decoction influenced the cell cycle of RA-FLS. (a, b) The cell cycle of RA-FLS was performed by flow cytometry. (c) The mRNA levels of cyclin D1 and CDK4 were tested by qRT-PCR. (d, e) The protein levels of cyclin D1 and CDK4 were tested by western blotting. ^∗^*P* < 0.05 compared with the normal group, ^#^*P* < 0.05 compared with the blank group; ^&^*P* < 0.05 compared with the P38 inhibitor group, and ^@^*P* < 0.05 compared with the PPAR*γ* agonist group.

As shown in Figures 4(a) and 4(b), the cell proportion in the S phase was notably increased while the G1 phase was notably decreased in the blank group contrasted with the normal group (all *P* < 0.05). In addition, the RA-FLS proportion in the S phase was remarkably reduced, whereas significantly elevated in the G1 phase in the P38 inhibitor group, PPAR*γ* agonist group, and Shentong Zhuyu Decoction group contrasted with the blank group (*P* < 0.05). However, cell proportion in the G2 phase was not obvious changes among different groups (*P* > 0.05). Furthermore, the results of qRT-PCR and western blotting indicated that the levels of cyclin D1 and CDK 4 in the blank group were higher than those in the normal group (*P* < 0.05), whereas P38 inhibitor, PPAR*γ* agonist, and Shentong Zhuyu Decoction suppressed the expressions of cyclin D1 and CDK4 (*P* < 0.05). Among the treatment groups in RA-FLS, the effects of P38 inhibitor on the expression of cyclin D1 and CDK4 were equivalent to the PPAR*γ* agonist (*P* > 0.05), while prior to the Shentong Zhuyu Decoction (*P* < 0.05) (Figures 4(c)–4(e)). All results suggested that Shentong Zhuyu Decoction inhibited the transition from the G1 phase to S phase of RA-FLS.

### 3.5. Shentong Zhuyu Decoction Inhibited Migration and Invasion of RA-FLS

**Figure 5:**
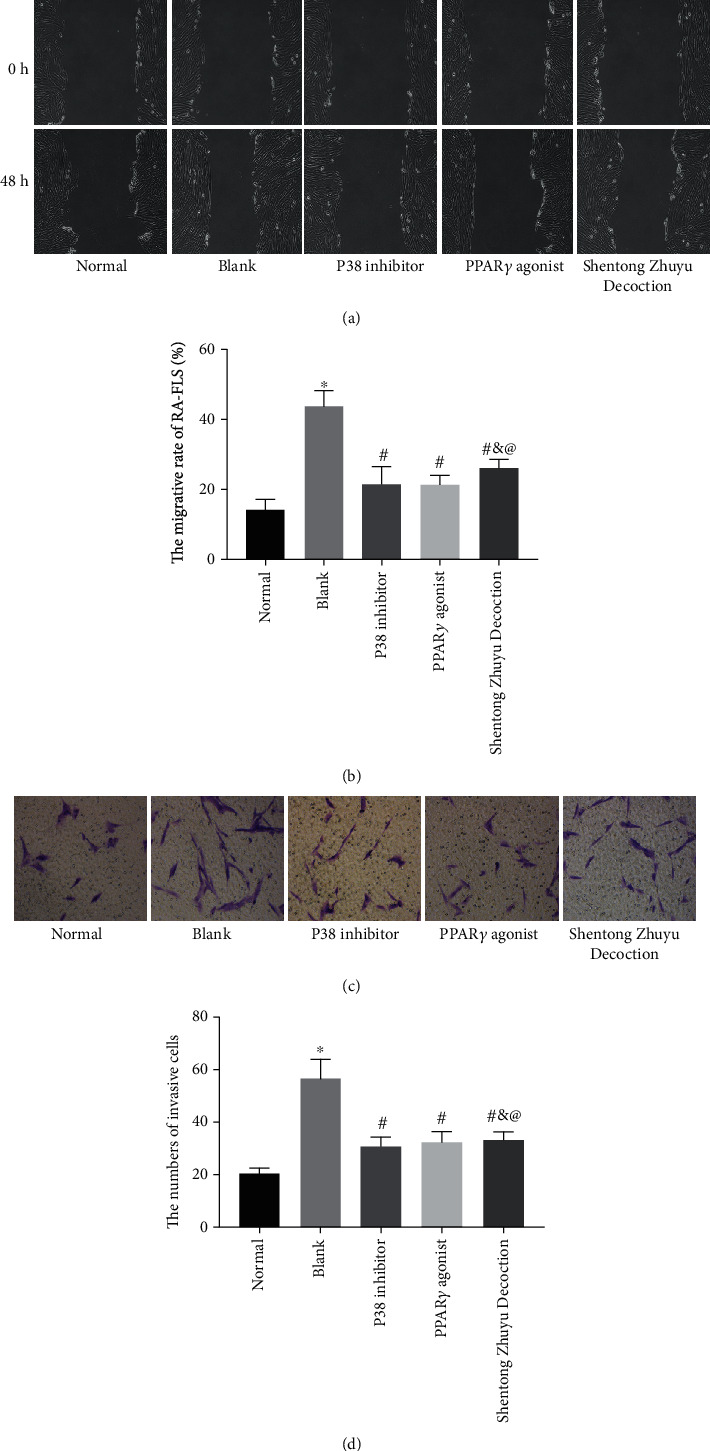
Shentong Zhuyu Decoction inhibited migration and invasion of RA-FLS. (a, b) The migration of RA-FLS was determined by the wound-healing assay (×100). (c, d) The numbers of invasion were determined by the transwell assay (×200). ^∗^*P* < 0.05 compared with the normal group, ^#^*P* < 0.05 compared with the blank group, ^&^*P* < 0.05 compared with the P38 inhibitor group, and ^@^*P* < 0.05 compared with the PPAR*γ* agonist group.

As shown in Figures 5(a) and 5(b), the wound-healing rate in the blank group was faster than that in the normal group (*P* < 0.05) and slower than that in the P38 inhibitor group, PPAR*γ* agonist group, and Shentong Zhuyu Decoction group (*P* < 0.05). Beyond that, the results of the transwell assay revealed that the numbers of invasive cells in the blank group were dramatically increased compared with the normal group, while were significantly decreased after intervened with P38 inhibitor, PPAR*γ* agonist, and Shentong Zhuyu Decoction (*P* < 0.05) (Figures 5(c) and 5(d)). Of these, the impact of P38 inhibitor and PPAR*γ* agonist on migration and invasion was stronger than Shentong Zhuyu Decoction (*P* < 0.05). Those findings suggested that Shentong Zhuyu Decoction inhibited the migration and invasion of RA-FLS.

### 3.6. Shentong Zhuyu Decoction Inhibited the Inflammatory Response of RA-FLS

**Figure 6:**
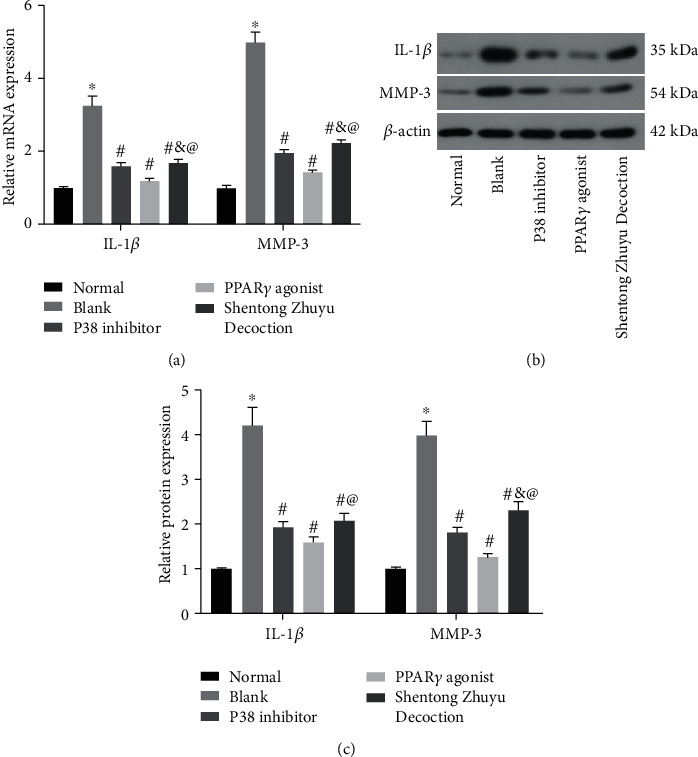
Shentong Zhuyu Decoction inhibited the inflammatory response of RA-FLS. (a) The mRNA levels of IL-1*β* and MMP3 were tested by qRT-PCR. (b, c) The protein levels of IL-1*β* and MMP-3 were tested by western blotting. ^∗^*P* < 0.05 compared with the normal group, ^#^*P* < 0.05 compared with the blank group, ^&^*P* < 0.05 compared with P38 inhibitor group, and ^@^*P* < 0.05 compared with the PPAR*γ* agonist group.

The results showed that the levels of IL-1*β* and MMP-3 in the blank group were obviously increased compared with the normal group (*P* < 0.05). When intervened with P38 inhibitor, PPAR*γ* agonist, and Shentong Zhuyu Decoction, the levels of IL-1*β* and MMP-3 were markedly reduced (*P* < 0.05). In addition, the effect of PPAR*γ* agonist on the IL-1*β* expression was stronger than P38 inhibitor and Shentong Zhuyu Decoction (*P* < 0.05). However, the MMP-3 expression in the Shentong Zhuyu Decoction group was higher than that in the P38 inhibitor group and PPAR*γ* agonist group (*P* < 0.05) (Figures 6(a)–6(c)).

### 3.7. Shentong Zhuyu Decoction Influenced the MAPK p38/PPAR*γ*/CTGF Pathway

**Figure 7:**
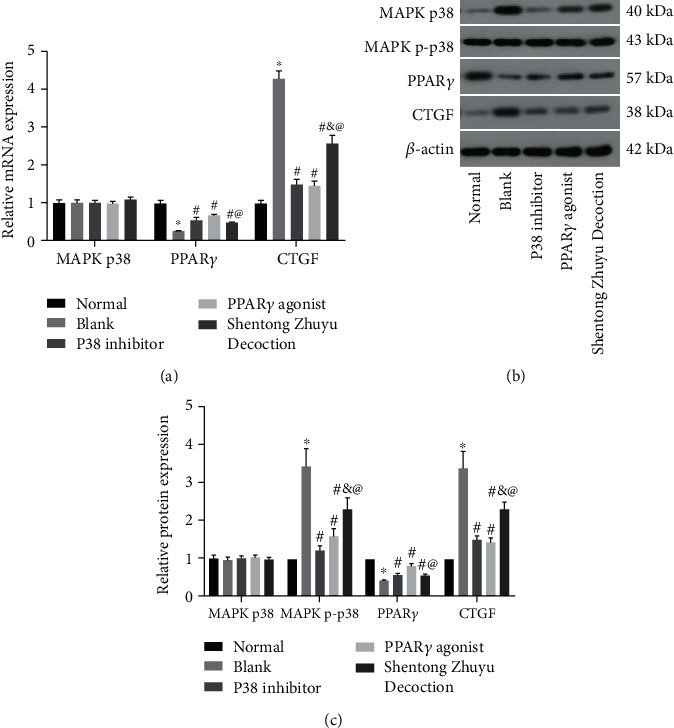
Shentong Zhuyu Decoction influenced the MAPK p38/PPAR*γ*/CTGF pathway. (a) The mRNA levels of MAPK p38, PPAR*γ*, and CTGF were tested by qRT-PCR. (b, c) The protein levels of MAPK p38, MAPK p-p38, PPAR*γ*, and CTGF were tested by western blotting. ^∗^*P* < 0.05 compared with the normal group, ^#^*P* < 0.05 compared with the blank group, ^&^*P* < 0.05 compared with the P38 inhibitor group, ^@^*P* < 0.05 compared with the PPAR*γ* agonist group.

In order to explain the different effects of P38 inhibitor, PPAR*γ* agonist, and Shentong Zhuyu Decoction on the RA-FLS, we investigated the expression of the MAPK p38/PPAR*γ*/CTGF pathway. As shown in Figures 7(a)–7(c), the mRNA and total protein levels of MAPK p38 were not obviously changes among the different groups (*P* > 0.05). Additionally, compared with the normal group, the levels of MAPK p-p38 and CTGF were markedly elevated, while the PPAR*γ* level was markedly reduced in the blank group (*P* < 0.05). Furthermore, P38 inhibitor, PPAR*γ* agonist, and Shentong Zhuyu Decoction all can reverse the expression of MAPK p-p38, PPAR*γ*, and CTGF in the RA-FLS (*P* < 0.05).

We also found that the MAPK p-p38 expression in the P38 inhibitor group was lower than that in the PPAR*γ* agonist group. In terms of the PPAR*γ* expression, the effect of PPAR*γ* agonist was higher than P38 inhibitor, whereas the level of CTGF was not obviously different in the two groups. So, we speculated that MAPK p-p38 may be upstream of PPAR*γ*, and CTGF may be jointly regulated by MAPK p-p38 and PPAR*γ*. In addition, the levels of MAPK p-p38 and CTGF were significantly decreased, and the PPAR*γ* level was notably elevated after intervention with Shentong Zhuyu Decoction in the RA-FLS (Figures 7(a)–7(c)). Abovementioned results indicated that Shentong Zhuyu Decoction may inhibit proliferation, migration, and invasion and promote apoptosis in the RA-FLS via regulating the MAPK p38/PPAR*γ*/CTGF pathway.

## 4. Discussion

RA pathogenesis is a systemic chronic inflammatory disease that is characterized as synovial cell proliferation with inflammatory infiltration, leading to joint damage [4]. RA-FLS not only play the role in the inflammatory secretion but also manifest tumor-like phenotype with high proliferation, migration, and invasion [29]. So, targeting RA-FLS is important for treatment for RA. Currently, the drugs are available including methotrexate, glucocorticoids, and ibuprofen, which may cause some side effects. Beyond that, some biological drugs including tocilizumab and tofacitinib are expensive for clinical treatment [30]. In past decades, traditional Chinese medicine explains the pathogenesis of RA and holds the treatment on the basis of symptoms and signs [20, 31]. Shentong Zhuyu Decoction is a classic representative formula for promoting blood circulation proposed by Qingren wang, which consists of 12 kinds of tradition Chinese herbs, including Gentiana macrophylla, Ligusticum wallichii, Peach Kernel, Safflower, Licorice, Notopterygium, Myrrh, Angelica, Trogopterus dung, Rhizoma Cyperi, Achyranthes, and Earthworm [21]. In the current study, the compound decoction added more 2 kinds of Chinese herbs on the basis of the above formula including Cortex phellodendri and Rhizoma Atractylodis to strengthen its power of dispelling wind, removing dampness, and clearing heat. Previous studies have proved that the combination of Cortex phellodendri and Rhizoma Atractylodis (Er-Miao-San) exhibits antiarthritic activity via regulating the balance of cytokines [32, 33]. The major finding of our study was that Shentong Zhuyu Decoction can inhibit the inflammatory response, migration, and invasion and promote apoptosis of rheumatoid arthritis fibroblast-like synoviocytes via the MAPK p38/PPAR*γ*/CTGF pathway.

Currently, induced by exogenous TNF-*α* and isolated from the synovial tissue are widely used for the RA model in vitro [34, 35]. In our study, the CIA rat model was established, and then RA-FLS were isolated and identified, as described previously [36]. The current study showed that the cells cultured for 3 generations were in accordance with the characteristics of RA-FLS. Additionally, we also found that the abilities of proliferation, migration, and invasion in the RA-FLS were elevated, while the apoptosis rate was reduced compared with normal FLS. Furthermore, a previous study has indicated that inducing apoptosis is deemed as the ideal therapy for RA patients [37]. As we all known, cell cycle consists of G1, G2, and S phase (interphase), and M phase (mitosis), which closely related to the cell proliferation and apoptosis. Moreover, cyclin D1 and CDK4 form a protein kinase complex, which participates in the regulation of restriction points in the G1 phase and then promotes cells transited to the S phase [38]. In terms of transition of the cell cycle in RA-FLS, Zhang et al. [39] have demonstrated that aspirin inhibits transition from the G1 phase to S phase and then promotes apoptosis and inhibits proliferation. Surprisingly, our study demonstrated that Shentong Zhuyu Decoction can suppress the transition from the G1 phase to S phase via inhibiting expressions of cyclin D1 and CDK4 in RA-FLS. Beyond that, we found that Shentong Zhuyu Decoction promoted the apoptosis rate of RA-FLS. In addition, the levels of Bax, Bcl-2, and caspase-3 confirmed the results of the apoptosis. The proteins (Bax, Bcl-2, and caspase-3) play crucial parts in apoptosis [31]. Abovementioned findings suggested that Shentong Zhuyu Decoction may become the targeting therapy for RA via regulating the cell biological behavior.

Inflammatory responses are the general event in RA patients, so antiinflammation therapy may be an effective treatment for relieving the symptoms [4]. It is well known that proinflammatory cytokines stimulate inflammatory responses and are considered as the key regulator in the progress of RA such as IL-1*β*, TNF-*α*, IL-6, and IL-17A [40]. In addition, these proinflammatory cytokines can promote the secretion of MMPs, which have been reported [41, 42]. Furthermore, Liu et al. [43] have indicated that Shentong Zhuyu Decoction can inhibit the secretion of inflammatory cytokines, attenuate cell infiltration, and improve state of joint. Surprisingly, the results of current study showed that the levels of IL-1*β* and MMP-3 were notably decreased after consumption of Shentong Zhuyu Decoction, which were consistent with previous report. Therefore, our study indicated that Shentong Zhuyu Decoction can effectively suppress the inflammatory response.

Until now, more evidences have verified that the MAPK signaling pathway involves in the regulation of inflammation and angiogenesis of RA [44–46] . Generally, MAPK p38 plays an important role on the secretion of proinflammatory cytokines [8]. A previous study has reported that MAPK p38 is upregulated and activated in RA-FLS [47]. Furthermore, Zou et al. have proved that SB203580 (p38 inhibitor) reverses the behaviors of activated RA-FLS [48]. Additionally, Ji et al. [49] have reported that pioglitazone (PPAR*γ* agonist) suppresses inflammation via blocking the MAPK p38 pathway. Subsequently, a similar study has indicated that pioglitazone can effectively reduce the expression of MAPK p38 [50]. Our study showed that the MAPK p-p38 expression in the P38 inhibitor group was lower than that in the PPAR*γ* agonist group, and the PPAR*γ* expression of the PPAR*γ* agonist group was higher than that of the P38 inhibitor group. So, we speculated that MAPK p38 may be upstream of PPAR*γ*, and there is a negative regulation relationship. CTGF is regarded as a novel mediator for cell growth, which was secreted by fibroblasts [51]. Apart from silencing of MAPK p38, it significantly decreases the CTGF expression, while PPAR*γ* agonist reduced the CTGF level [52, 53]. In our study, the CTGF level was not obviously different between the P38 inhibitor group and PPAR*γ* agonist group. Those findings indicated that CTGF may be jointly regulated by MAPK p-p38 and PPAR*γ*. Furthermore, we also found that Shentong Zhuyu Decoction inhibited the levels of MAPK p-p38 and CTGF and promoted the PPAR*γ* level. The results of Shentong Zhuyu Decoction were consistent with the trends of SB203580 and GW1929. Therefore, abovementioned results suggested that Shentong Zhuyu Decoction regulated the behaviors of RA-FLS via the MAPK p38/PPAR*γ*/CTGF pathway.

The lack of research on Shentong Zhuyu Decoction in vitro is a limitation of this study. So, we will explore the effect of Shentong Zhuyu Decoction on the CIA model and verify the effect of drug-containing serum via administration of Shentong Zhuyu Decoction in vivo in the further.

## 5. Conclusions

The current study demonstrated that Shentong Zhuyu Decoction inhibited inflammatory response, transition from G1 phase to S phase, migration, and invasion and promoted apoptosis of RA-FLS via regulating the MAPK p38/PPAR*γ*/CTGF pathway.

## Data Availability

All data generated or analyzed in this study are included in this published article.
